# Association between inflammatory hematological ratios and aortic valve stenosis: a propensity-matched analysis

**DOI:** 10.3389/fcvm.2025.1482313

**Published:** 2026-01-12

**Authors:** Xin Gong, Min Wang, Liang Wang, Shangwei Huang, Bijun Huang, Tian Chen, Lianghua Xia, Wei Wei, Qi Zhang, Jianqiang Hu, Jing Xu

**Affiliations:** 1 Department of Cardiology, Shanghai East Hospital, Shanghai, Tongji University School of Medicine, Shanghai, China; 2 Department of General Medicine, Beicai Community Health Service Center of Pudong New District, Shanghai, China; 3 Department of Ultrasonography, Shanghai East Hospital, Tongji University School of Medicine, Shanghai, China

**Keywords:** aortic valve stenosis, cholesterol, inflammation hematological ratios, monocyte/high density lipoprotein ratio, propensity score matching analysis

## Abstract

**Background:**

Relationship between systemic inflammation and aortic valve stenosis (AVS) has been well demonstrated. This investigation aimed to evaluate the link between various inflammation hematological ratios and patients with AVS.

**Methods:**

Patients with AVS (*n* = 229) and control (*n* = 1716) were identified. Propensity score matching (PSM) was performed with the proportion of 1:1 using logistic regression based on the variables of age, sex, hypertension, diabetes, creatinine (Cr) and glutamate transpeptidase (γ-GT). The receiver operating characteristic (ROC) analysis was used to estimate the performance of inflammation hematological ratios for distinguishing AVS. Univariate and multivariate analysis were performed to identify the independent risk factors of AVS. In addition, restricted cubic splines (RCS) regression analysis revealed the non-linear correlation between inflammation hematological ratios and AVS.

**Results:**

After PSM, 392 patients (196:196) were included. Univariate analysis showed monocyte/high density lipoprotein ratio (MHR) and neutrophil/lymphocyte ratio (NLR) were significantly higher in AVS group (MHR, 0.49 ± 0.23 vs 0.32 ± 0.20, *p* < 0.001, NLR, 3.52 ± 2.52 vs 2.87 ± 1.89, *p* < 0.001) while lymphocyte/monocyte ratio (LMR) was significantly lower (3.27 ± 1.72 vs 4.08 ± 1.76, *p* < 0.001). But the level of platelet/lymphocyte ratio (PLR) and systemic inflammation index (SII) (PLR, 146.27 ± 82.68 vs 143.27 ± 66.95, *p* = 0.927, SII, 690.22 ± 602.69 vs 610.58 ± 403.33, *p* = 0.100) did not differ significantly between the two groups. However, in multivariate logistic regression models, only MHR remained to be an independent risk factor of AVS (OR 2.010 with 95%CI 1.040-3.887, *p* = 0.038). Besides MHR, atrial fibrillation (AF), low-density lipoprotein cholesterol (LDL-C), high-density lipoprotein cholesterol (HDL-C), triglycerides (TG) and hemoglobin (Hgb) were also performed to be independent risk factors of AVS. ROC analysis showed that the cut-off value of MHR (0.2750) indicated AVS with 85.2% sensitivity and 51.5% specificity [95% confidence interval [CI] = 0.691–0.788, the area under the curve [AUC] = 0.740, *P* < 0.001]. Further, RCS revealed the non-linear correlation between MHR and AVS (p for non-linearity <0.001).

**Conclusions:**

Elevated MHR was independently associated with the presence of AVS. MHR demonstrated stronger associational strength with AVS compared to other inflammatory hematological ratios such as SII, NLR, PLR and LMR.

## Introduction

Aortic valve stenosis (AVS) is one of the most common cardiovascular diseases with considerable impact on morbidity and mortality. AVS is progressive with age and present in 2%–5% of patients aged over 65 years (1–3). Still, recent data indicate that chronic inflammation and lipid metabolism disorders play pivotal roles in fibrosis formation and leaflet thickening, which results in severe AVS (4–7).

Over the past decades, close interaction of inflammation and immune system-related cells such as neutrophils, platelets and lymphocyte with the pathogenesis of AVS has attracted great attention (8–12). Accordingly, various inflammation hematological ratios have been developed from the count of these cells in diverse combinations in order to predict the progression and prognosis of AVS (8, 10, 11, 13). Among them, neutrophil/lymphocyte ratio (NLR), platelet/lymphocyte ratio (PLR) and lymphocyte/monocyte ratio (LMR) were well-defined inflammation biomarkers that were found to be associated with the severity of AVS in previous studies (10, 11, 14). Systemic inflammation index (SII) was also a novel marker that brought together these peripheral cell counts of neutrophils, lymphocytes, and platelets, and was demonstrated to predict severe calcific AVS (9). In a recent study, monocyte/high density lipoprotein ratio (MHR) was determined as a significant independent predictor for the speed of progression and diagnosis of severe bicuspid AVS (15). However, there is debate about which of the above inflammatory hematological ratios has the best predictive performance for AVS risk.

This study sought to investigate the association between various inflammatory hematological ratios and AVS.

## Methods

### Study population

**Figure 1:**
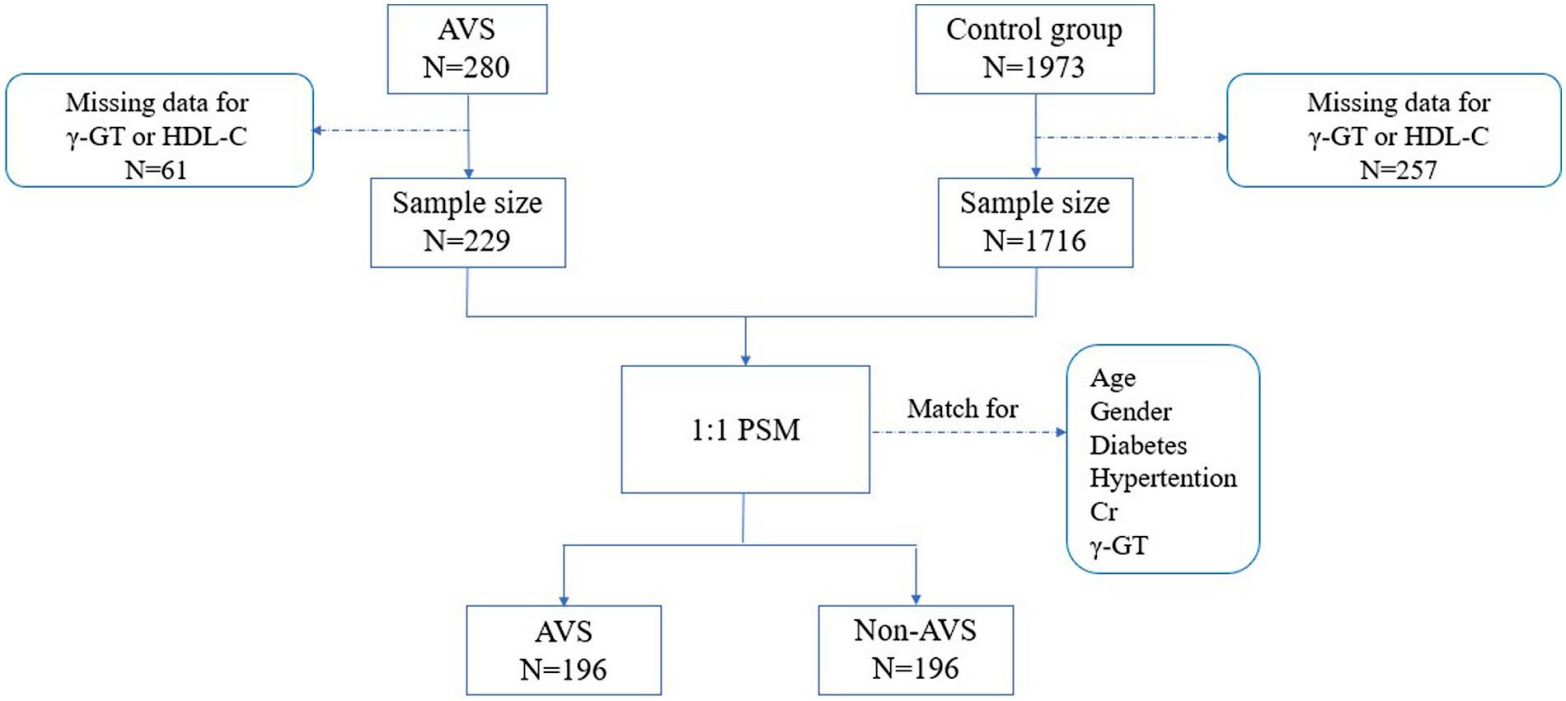
A flow chart of the patient enrollment. AVS, aortic valve stenosis group; non-AVS, control group; γ-GT, glutamate transpeptidase; HDL-C, high-density lipoprotein cholesterol; Cr, creatinine.

This was a retrospective, observational study. We screened consecutive patients who underwent transthoracic echocardiography (TTE) at the Heart Center of Shanghai East Hospital between September 2019 and September 2023. Patients were included based on the availability of complete laboratory blood tests and echocardiographic data. Exclusion criteria were: no echocardiographic evaluation, white blood cells >11 × 10^9^/L, creatinine (Cr) >707 µmol/L, history of aortic valve replacement surgery, infective endocarditis, acute myocardial infarction, history of immune system disease, and severe aortic valve regurgitation. All patients were evaluated by TTE and categorized into the aortic valve stenosis group (AVS group) or the control group (Non-AVS group). Patients in the control group were further excluded if they had any structural or functional cardiac abnormalities. All data were retrospectively collected from the electronic medical record system (Figure 1).

### Laboratory measurements

Laboratory data were retrieved retrospectively from the electronic medical records.Total complete blood count test (Sysmex K-1000; Sysmex Corporation, Kobe, Japan) and blood chemistry parameters (Modular Systems; Roche Diagnostics, Tokyo, Japan) were carried out at the biochemistry laboratory during their prior hospital visits between September 2019 and September 2023.

Inflammation hematological ratios were calculated using the following formulas: MHR (monocyte/high density lipoprotein ratio) = monocyte count ÷ HDL-cholesterol value. LMR (lymphocyte/monocyte ratio) = lymphocyte count÷ monocyte count. NLR (neutrophil/lymphocyte ratio) = neutrophil count ÷ lymphocyte count; PLR (platelet/lymphocyte ratio) = platelet count ÷ lymphocyte count; SII (systemic inflammation index) = neutrophil count × platelet count ÷ lymphocyte count.

### Echocardiographic examination

Echocardiographic data were obtained retrospectively from stored examinations performed during routine clinical care. Echocardiographic evaluation was obtained using a Philips Epiq7C machine (Phillips, IE) at the Department of Ultrasound in Shanghai East Hospital. The echocardiographers were blind to clinical information. Standard Doppler parameters including ejection fraction, left ventricular end diastolic diameter, aortic maximal jet velocity, and aortic valve maximum pressure gradient were recorded. The diagnosis for AVS met the American College of Cardiology/American Heart Association standards (16). AVS was defined as leaflet calcification and a transaortic peak velocity >2.5 m/s by continuous-wave Doppler.

### Statistical analysis

Continuous variables were presented as median with interquartile ranges (IQRs) and were tested normal distribution by one sample Kolmogorov–Smirnov test. Categorical variables were presented as numbers and percentages. Normally distributed continuous variables were tested by independent-sample *t* test while abnormally distributed continuous variables were tested by Mann–Whitney *U* test. Categorical variables were compared by the Chi-square test.

We used receiver operating characteristic (ROC) analysis to estimate the performance of inflammation hematological ratios for distinguishing AVS. The optimal cut-off value was calculated based on the highest Youden index (sensitivity + specificity—1). Two logistic regression models were performed to analysis the association between AVS and inflammation hematological ratios. The variables which were considered to be significantly different in univariate analysis were included in Model 2 to adjust the performance of inflammation hematological ratios. Restricted cubic splines (RCS) regression analysis was used to explore the non-linear correlation between inflammation hematological ratios and AVS. Four knots at the 5th, 35th, 65th and 95th percentiles were placed in RCS regression analysis.

*P* < 0.05 was considered as statistically significant in all of the tests. All the statistical analysis was performed with IBM SPSS Statistics, Version 25.0 (IBM Corp., Armonk, New York, USA) and R, Version 4.2.1 (R Foundation for Statistical Computing, Vienna, Austria).

### Propensity score matching analysis

Two hundred and twenty-nine AVS patients and 1,716 Non-AVS patients were enrolled in this study. Due to the differences of baseline and sample size between the AVS group and control group, propensity score matching (PSM) was performed with the proportion of 1:1. The propensity score was evaluated using logistic regression based on the variables of age, sex, hypertension, diabetes, creatinine (Cr) and glutamate transpeptidase (γ-GT). The Match Tolerance was set to 0.02. Absolute standard differences were used to assess the ability of the matching. The matched patients were included in the following analysis.

## Results

### Characteristics and echocardiographic parameters

**Figure 2:**
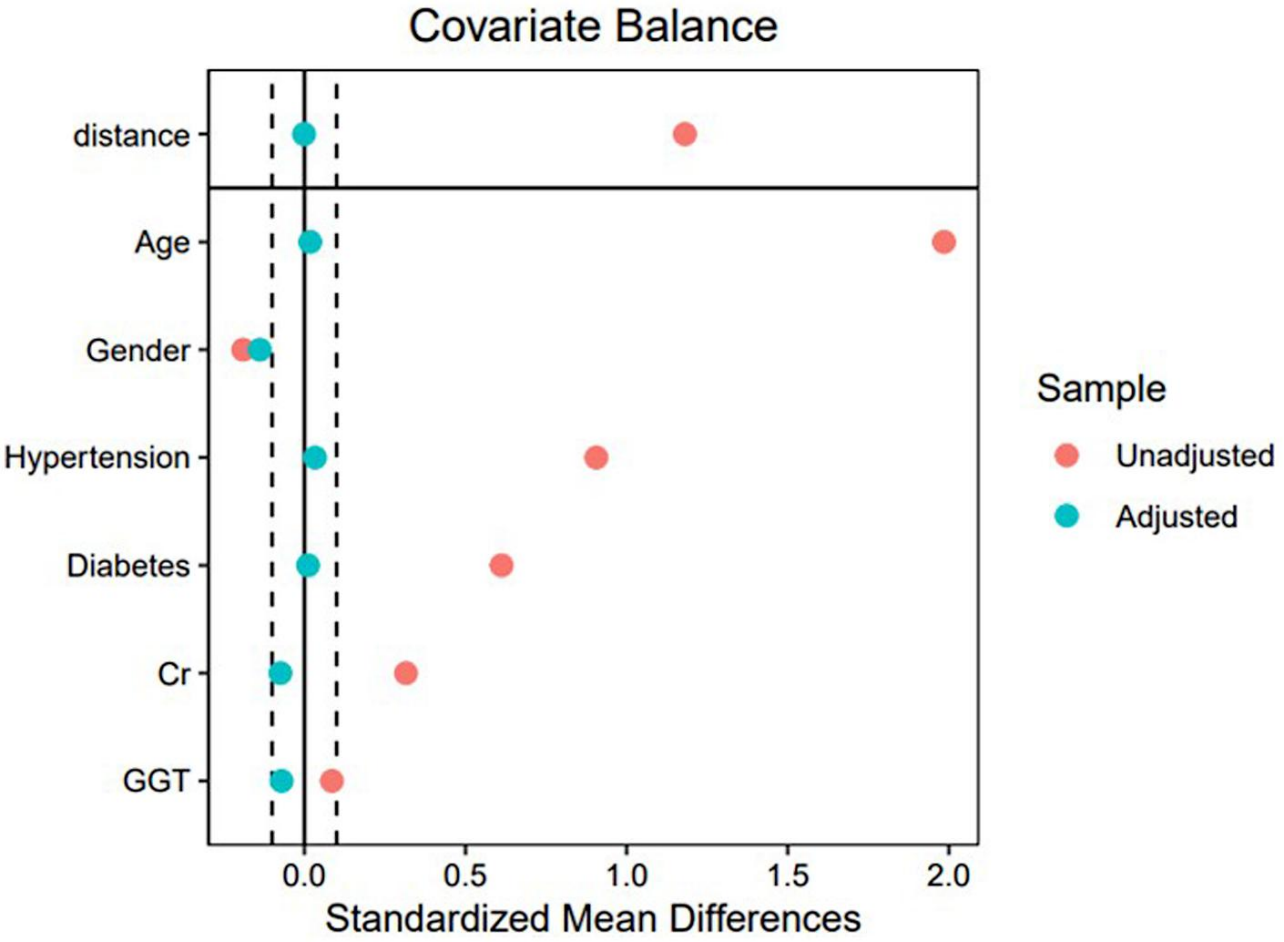
Love plots for baseline covariates between AVS and control group, before and after propensity score matching. GGT, glutamate transpeptidase; Cr, creatinine.

After PSM, 196 patients with AVS and 196 patients without AVS were included. Figure 2 showed all the measured covariates before and after PSM, absolute standardized differences for all measured covariates were <10% after matching. Age, sex, hypertension' diabetes, Cr and '-GT were similar in the matched group (*p* > 0.05, Table 1).

**Table 1:** Characteristics and echocardiographic parameters in patients with AVS and control group.

Variables	Overall, *n* = 392	AVS, *n* = 196	Non-AVS, *n* = 196	*P*
Variables for PSM				
Age, y, median (IQR)	77 (69–83)	77 (70–83)	79 (68–83)	0.492
Male, *n* (%)	202 (51.5)	99 (50.5)	103 (52.6)	0.744
Hypertension, *n* (%)	328 (83.7)	164 (83.7)	164 (83.7)	0.974
Diabetes, *n* (%)	155 (39.5)	75 (38.3)	80 (40.8)	0.580
*γ*-GT, U/L, median (IQR)	23 (16.03–36.75)	25.85 (17.10–45.30)	21.00 (16.00–30.00)	0.763
Cr, umol/L, median (IQR)	78.50 (65.05–95.75)	76.80 (62.00–96.90)	80.00 (68.00–95.00)	0.911
Medical history				
Atrial fibrillation, *n* (%)	87 (22.2)	61 (31.1)	26 (13.3)	0.000
Laboratory parameters				
WBC, × 10^9^/L, median (IQR)	6.48 (5.58–7.72)	6.60 (5.63–7.84)	6.32 (5.54–7.50)	0.103
PLT, ×10^9^/L, median (IQR)	199.50 (160.00–247.75)	195.50 (149.25–245.75)	208.50 (176.25–249.50)	0.025
Hgb, g/L, median (IQR)	130.50 (117.00–143.00)	127.00 (111.25–142.75)	132.00 (120.25–144.75)	0.001
Lym, ×10^9^/L, median (IQR)	1.57 (1.14–1.96)	1.53 (1.10–1.85)	1.66 (1.17–2.07)	0.027
Neu, ×10^9^/L, median (IQR)	4.12 (3.31–5.16)	4.23 (3.41–5.48)	4.02 (3.19–4.96)	0.014
Mon, ×10^9^/L, median (IQR)	0.46 (0.37–0.58)	0.51 (0.39–0.63)	0.43 (0.35–0.53)	0.000
HDL-C, mmol/L, median (IQR)	1.24 (1.01–1.63)	1.14 (0.96–1.38)	1.47 (1.10–2.40)	0.000
TC, mmol/L, median (IQR)	3.96 (3.21–4.77)	3.74 (3.12–4.59)	4.16 (3.39–5.05)	0.001
TG, mmol/L, median (IQR)	1.19 (0.94–1.69)	1.12 (0.84–1.58)	1.30 (1.00–1.81)	0.003
LDL-C, mmol/L, median (IQR)	1.90 (1.33–2.82)	2.16 (1.64–2.87)	1.63 (1.16–2.74)	0.000
HbAlc, %, median (IQR)	6.3 (5.8–7.2)	6.3 (5.8–7.3)	6.2 (5.7–7.2)	0.751
FPG, mmol/L, median (IQR)	5.67 (4.98–6.86)	5.52 (4.88–7.08)	5.84 (5.18–6.80)	0.053
Echocardiographic measurement				
EF, %, median (IQR)	65 (61–68)	64 (60–67)	66 (63–68)	0.000
LA, mm, median (IQR)	38 (35–42)	41 (37–44)	36 (34–40)	0.000
LVPW, mm, median (IQR)	9 (9–10)	10 (9–11)	9 (9–10)	0.000
IVS, mm, median (IQR)	9 (10–11)	10 (9–11)	9 (9–10)	0.000
LVDd, mm, median (IQR)	45 (42–49)	46 (43–50)	45 (41–48)	0.000
LVSd, mm, median (IQR)	29 (27–31)	29 (27–30)	28 (26–30)	0.000
Peak aortic transvalvular velocity, m/s, median (IQR)	/	2.9 (2.7–3.4)	/	
Mean aortic valve gradient, mmHg, median (IQR)	/	19(15–27)	/	

Categorical variables are shown as number (percentage) and continuous variables as median (IQR).

AVS, aortic valve stenosis group; non-AVS, control group; IQR, interquartile range; γ-GT, glutamate transpeptidase; Cr, creatinine; WBC, white blood cell; PLT, blood platelet; Hgb, hemoglobin; Lym, lymphocyte; Neu, neutrophils; Mon, monocytes; HDL-C, high-density lipoprotein cholesterol; TG, triglycerides; TC, total cholesterol; LDL-C, low-density lipoprotein cholesterol; HbAlc, glycosylated hemoglobin; FPG, fasting plasma glucose; EF, ejection fraction; LA, left atrium; LVPW, left ventricular posterior wall; IVS, interventricular septum; LVDd, left ventricular end diastolic diameter; LVSd, left ventricular end systolic diameter.

Table 1 showed the characteristics of the matched groups. Compared with the control group, the AVS group had a higher level of neutrophils (Neu), monocytes (Mon), high-density lipoprotein cholesterol (HDL-C), low-density lipoprotein cholesterol (LDL-C), total cholesterol (TC), triglycerides (TG) while a lower level of lymphocyte (Lym), hemoglobin (Hgb) and blood platelet (PLT) (*p* < 0.05). In addition, the two groups differed significantly with the echocardiographic parameters and the proportion of atrial fibrillation (AF) (*p* < 0.001). White blood cell (WBC), glycosylated hemoglobin (HbAlc), and fast blood glucose didn't differ significantly between the two groups (*p* > 0.05).

### Inflammation hematological ratios in AVS and non-AVS

**Figure 3:**
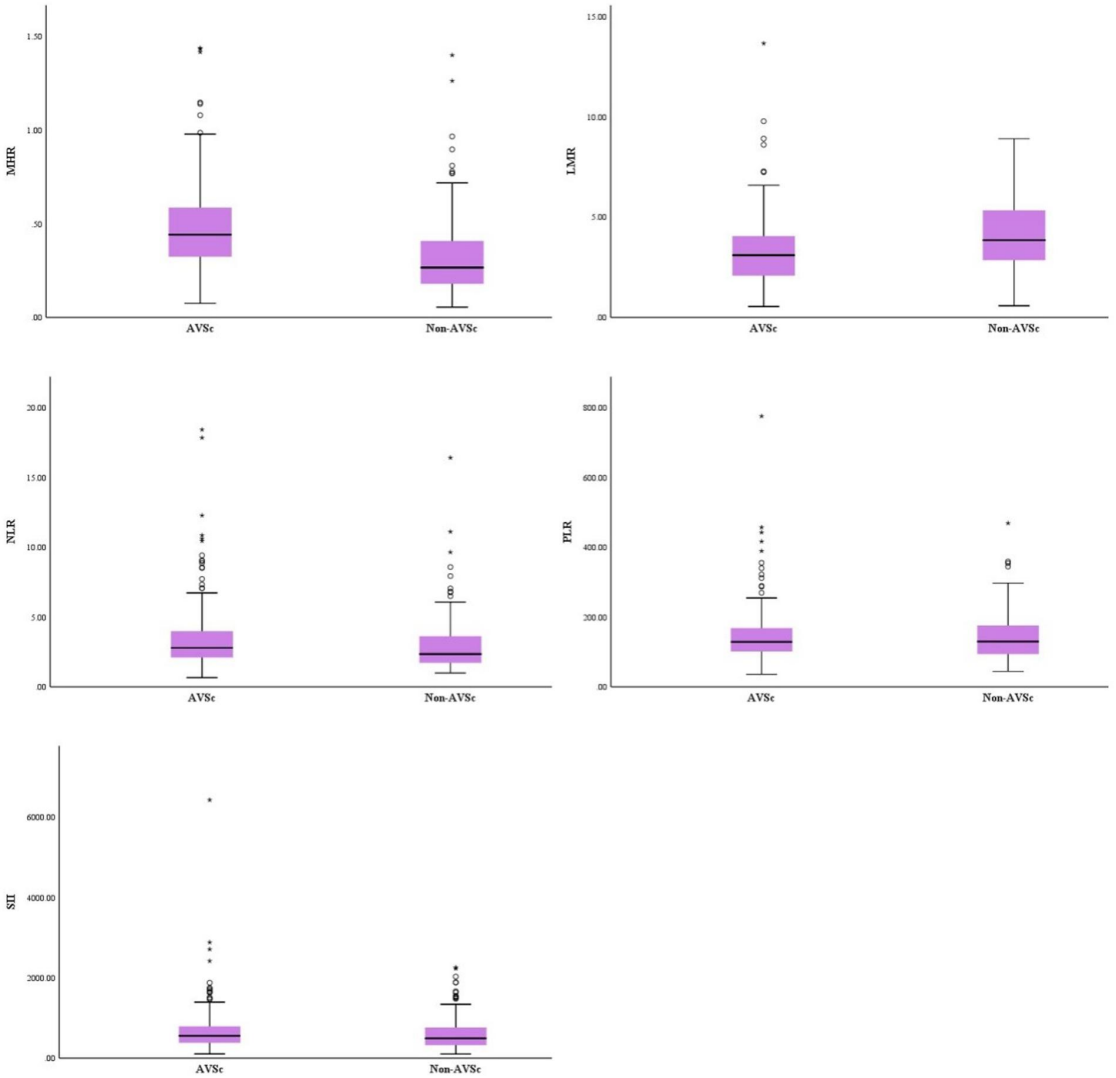
Comparison of inflammation hematological ratios between patients with and without AVS.

**Figure 4:**
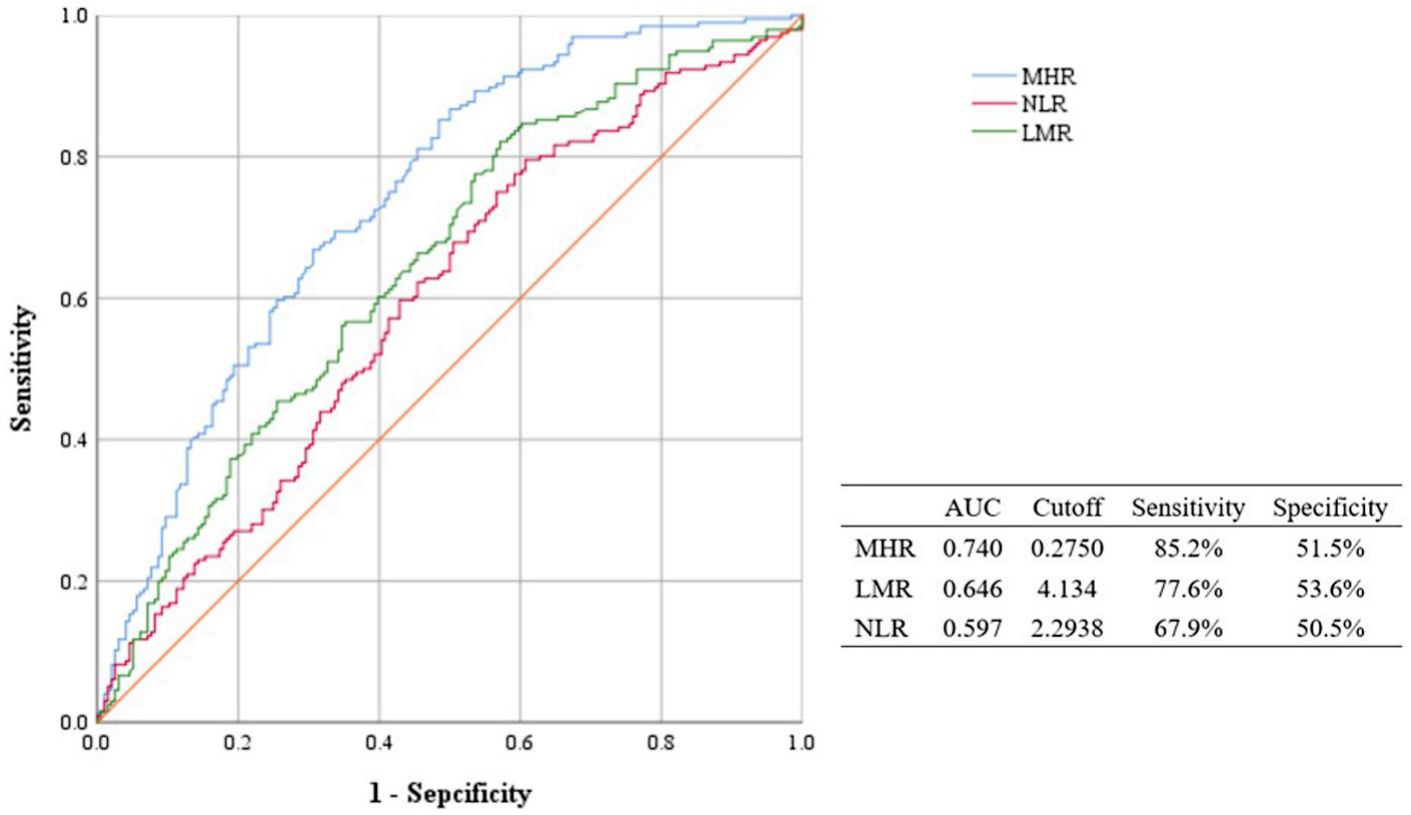
Roc curves and cut-off of MHR, NLR and LMR for AVS. MHR, monocyte/high density lipoprotein ratio; LMR: lymphocyte/monocyte ratio; NLR, neutrophil/lymphocyte ratio; AUC, area under curve.

Figure 3 showed the differences of inflammation hematological ratios between the two groups. MHR and NLR were significantly higher in AVS group (MHR, 0.49 ± 0.23 vs 0.32 ± 0.20, *p* < 0.001, NLR, 3.52 ± 2.52 vs 2.87 ± 1.89, *p* < 0.001). LMR in AVS patients was significantly lower than the Non-AVS group (3.27 ± 1.72 vs 4.08 ± 1.76, *p* < 0.001). The two groups had the similar level of PLR and SII (PLR, 146.27 ± 82.68 vs 143.27 ± 66.95, *p* = 0.927, SII, 690.22 ± 602.69 vs 610.58 ± 403.33, *p* = 0.100). The cut-off value of MHR (0.2750) indicated AVS with 85.2% sensitivity, 51.5% specificity, [95% confidence interval (CI) = 0.691–0.788, AUC=0.740, *p* < 0.001]. DeLong tests showed MHR's AUC was significantly higher than LMR and NLR (MHR vs LMR, *p* < 0.001; MHR vs NLR, *p* < 0.001; LMR vs NLR, *p* < 0.001). Details of ROC analysis were displayed in Figure 4.

### Multivariate analysis of inflammation hematological ratios with AVS

Table 2 displayed the results of multivariate logistic regression analysis validating factors associated with AVS. Models 1 demonstrated MHR (OR 5.870 with 95%CI 3.585–9.612, *p* < 0.001) was an independent risk factor of AVS. After adjusting for other risk factors in model 2, MHR (OR 2.010 with 95%CI 1.040–3.887, *p* = 0.038) was still found to be significant as the independent risk factor of AVS. Besides MHR, AF, LDL-C, TG and Hgb were performed to be independent risk factors of AVS in model 2. LMR and NLR were evaluated but not selected in the final model.

**Table 2:** Multivariate logistic regression analysis for the presence of AVS.

Independent variables	Model 1			Model 2	
	Odds ratio (95% CI)	*P* value		Odds ratio (95% CI)	*P* value
MHR	5.870 (3.585–9.612)	0.000		2.010 (1.040–3.887)	0.038
LMR	0.609 (0.347–1.070)	0.085		0.605 (0.324–1.132)	0.116
NLR	1.517 (0.857–2.686)	0.153		1.235 (0.666–2.287)	0.530
Atrial fibrillation				2.889 (1.543–5.445)	0.001
HDL-C				0.202 (0.104–0.392)	0.000
LDL-C				1.351 (1.037–1.760)	0.026
TG				0.581 (0.418–0.806)	0.001
Hgb				0.978 (0.964–0.991)	0.001

MHR, monocyte/high density lipoprotein ratio; LMR: lymphocyte/monocyte ratio; NLR, neutrophil/lymphocyte ratio; LDL-C, low-density lipoprotein cholesterol; HDL-C, high-density lipoprotein cholesterol; TG, triglycerides; Hgb, hemoglobin.

### RCS of inflammation hematological ratios with AVS

**Figure 5:**
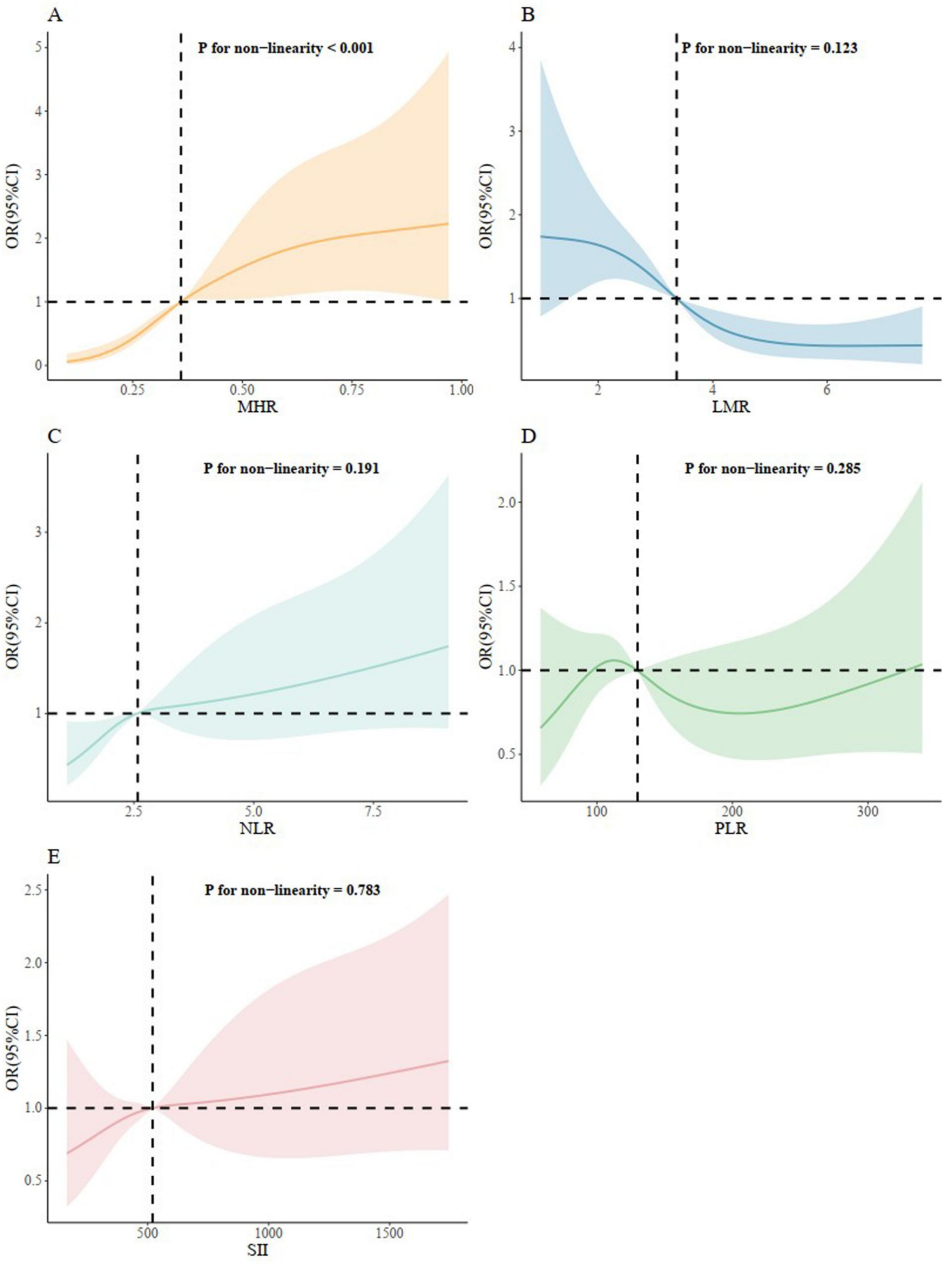
Restricted cubic splines regression analysis of inflammation hematological ratios with AVS. **(A)** for MHR; **(B)** for LMR; **(C)** for NLR; **(D)** for PLR; **(E)** for SII. MHR, monocyte/high density lipoprotein ratio; LMR, lymphocyte/monocyte ratio; NLR, neutrophil/lymphocyte ratio; PLR, platelet/lymphocyte ratio; SII, systemic inflammation index.

We further conducted the RCS to evaluated the correlation between inflammation hematological ratios and the risk of AVS (Figure 5). A nonlinear and S-shaped association was showed between MHR and AVS (*p* for non-linearity <0.001) in the model. Segmented regression identified two breakpoints at 0.33 and 0.83, while the OR = 1 reference corresponded to MHR = 0.36. There was no nonlinear association between LMR, NLR, PLR, SII and AVS.

## Discussion

In this retrospective cross-sectional study, we demonstrated that MHR level was significantly increased in AVS patients compared to patients without AVS, indicating MHR was significantly associated with the presence of AVS. Furthermore, multivariable analysis revealed a stronger independent association between MHR and the presence of AVS compared to established inflammatory ratios such as SII, NLR, PLR, and LMR. To our knowledge, this is one of the few studies to comprehensively compare multiple inflammatory hematological ratios, including MHR, SII, NLR, PLR, and LMR, in relation to AVS using PSM to control for confounding factors. Our findings highlight the superior association of MHR with AVS compared to other established ratios.

AVS is a progressive valvular disease in the population that resembles atherosclerosis with activation of calcification, lipoprotein deposition and chronic inflammation (17–20). The endothelial damage resulting from increased mechanical stress and reduced shear stress may allow lipids to penetrate the valvular endothelium, and subsequently, lipid deposition and oxidation occur in valvular endothelium. Afterward, complex inflammatory pathways are activated with the contribution of inflammatory and immune system-related cells such as monocytes, macrophages, and lymphocytes (21, 22). Biomarkers derived from the counts of the cell types have been widely investigated in recent years due to the fact that they are affordable and available. Among them, PLR, NLR and LMR were shown to possess predictive and prognostic roles in AVS (8, 10, 14). A previous study demonstrated that NLR was related to the severity of calcific AVS and LV systolic dysfunction in patients with severe calcific AVS (23–25). Likewise, PLR was shown to been independently associated with the presence of AVS (11, 25). Other studies have also proven that increased PLR was linked with the severity of degenerative AVS, and PLR should be used to monitor patients' inflammatory responses and the efficacy of treatment (25, 26). SII, which include the peripheral counts of neutrophils, lymphocytes, and platelets, was recently found to be better than NLR and PLR in predicting severe calcific AVS (9, 27). However, it is not clear which indicator has the most excellent correlation with AVS. In our study, we included MHR, a novel inflammatory marker, and focused on the comparison on AVS association ability among diverse inflammatory hematological ratios. The results highlighted that MHR emerged stronger associational strength with AVS compared to other inflammatory hematological ratios such as SII, NLR, PLR and LMR. More importantly, compared to previous studies, we used propensity score matching analysis to balance the baseline confounding factors that may affect the incidence of AVS, including age, gender, hypertension, diabetes, Cr and *γ*-GT, partly minimizing the impact of selection bias on the results.

Consistent with the results of our study, a significant association between MHR levels and inflammation was demonstrated by the study of Acikgoz et al. (28). Monocytes are pivotal immune cells and molecules that associate with endothelial cells, attending to aggravation of inflammatory pathways. Since macrophages originate from circulating monocytes, the number of circulating monocytes and monocyte subsets with different properties has attracted attention in both atherosclerosis lesions and valvular disease studies (15, 29, 30). Moreover, HDL-cholesterol is well-known as an anti-inflammatory indicator. HDL protects endothelial cells from inflammation and oxidative stress by preventing the displacement of macrophages and oxidation of low-density lipoprotein molecules, and also by controlling monocyte progenitor cell proliferation and monocyte activation (31–33). Therefore, it is reasonable to use MHR as a single marker by integrating monocyte and HDL-C. The advantage of MHR is that it provides more reliable information than either monocyte or lipoprotein alone for predicting inflammatory and cholesterol burden. Recently, MHR has been reported to be a novel marker for valvular heart disease (34–38). Ozcan et al. reported that MHR is strongly associated with mitral valve prolapse (MVP) and might be a prognostic factor for patients with MVP (34). In our study, ROC analysis and further DeLong tests showed that MHR indicated AVS with a higher sensitivity than LMR and NLR. Most notably, among the five inflammation hematological ratios evaluated, only MHR was positively associated with AVS after adjusting for the confounding factors. Furthermore, in comparison to previous studies, a nonlinear and S-shaped association was firstly revealed between MHR and AVS using RCS analysis while no nonlinear association were shown between other inflammation hematological ratios and AVS, providing more meaningful evidence for the close relationship between MHR and AVS. RCS analysis showed risk accelerated once MHR exceeded 0.33 and plateaus after 0.83, supporting 0.33 as an early-intervention threshold and 0.83 as a high-risk ceiling in clinical practice.

The precise role of the MHR in the pathogenesis of AVS has yet to be fully elucidated. Monocytes and their differentiated descendants, macrophages, play a central role in the initiation and progression of aortic valve calcification (39). In the early stages of AVS, mechanical stress and endothelial dysfunction facilitate the infiltration of lipids and circulating monocytes into the valvular subendothelium. Once localized, monocytes differentiate into macrophages, which phagocytose oxidized lipids and transform into foam cells—a key feature of early valve lesions. These activated macrophages secrete pro-inflammatory cytokines (e.g., TNF-α, IL-1β, IL-6) and promote the expression of osteogenic mediators such as bone morphogenetic protein-2 (BMP-2) and alkaline phosphatase, thereby driving the transition from inflammation to active calcification (40, 41). HDL cholesterol exerts atheroprotective and anti-inflammatory effects that counter these processes. Functionally intact HDL facilitates reverse cholesterol transport from macrophage foam cells, reduces lipid peroxidation, and inhibits the expression of adhesion molecules on endothelial cells, thereby limiting further monocyte recruitment (42). Moreover, HDL-associated enzymes like paraoxonase-1 attenuate oxidative stress, while HDL modulates monocyte activation and macrophage polarization toward anti-inflammatory phenotypes (43). In AVS, however, HDL may become dysfunctional—losing its protective capacity—due to oxidative modification or systemic inflammation, thereby exacerbating valvular injury and calcification (15). The MHR thus integrates both the pro-inflammatory burden (monocyte activation) and the anti-inflammatory capacity (HDL function), offering a composite biomarker that reflects the net inflammatory–lipid imbalance driving valvular calcification.

TG and LDL are also identified as associated factors of AVS in addition to MHR in multivariate logistic regression analysis. The pathophysiology of AVS was consisted of both an initiation phase including lipid infiltration, oxidation and inflammation, and a propagation phase characterized by fibrosis and calcification. Emerging studies revealed lifelong exposure to high cholesterol increases the risk of symptomatic AVS (19, 44, 45). Additionally, the prevalence of AF in patients with AVS is high and AF is associated with poor prognosis in AVS (46–48). Age-related changes in heart structure (left atrial dilation, left ventricular hypertrophy and increased fibrosis) in conjunction with chronic pressure or volume overload induced by the valvular obstacle may explain this over-representation of AF in AVS.

Our study has important clinical relevance. In this study, we confirmed a statistically significant correlation between increased MHR and AVS. These results are consistent with the previous evidence that inflammation and cholesterol play essential roles in the pathology of AVS. Accordingly, modulation of inflammation with the anti-inflammatory therapies combined with statin may slow the progression of AVS. Furthermore, MHR can be calculated easily from the routine blood parameters, and also MHR should be considered as an early associated factor of patients who have a high inflammatory risk and a high rate of progression to severe AVS.

### Study limitations

Our study has several limitations. Firstly, this study is a retrospective and observational study with a relatively limited number of patients. We did not collect longitudinal follow-up information on AVS progression in this cohort, and thus cannot assess the predictive or monitoring value of these inflammatory ratios for disease progression over time. Secondly, other inflammatory markers such as C-reactive protein and interleukin-6 were not evaluated in this study. Thirdly, we measured inflammation hematological ratios only at baseline rather than investigation of temporal variations. Finally, although we carefully controlled for the major known confounders, unknown factors may still have interfered inflammation parameters. A large number of large-scale, multicenter prospective studies with serial measurements are still needed in the future to further illustrate the predictive value of MHR in patients with AVS and evaluate their role in tracking AVS progression.

## Conclusions

The present study suggested that elevated MHR level was significantly associated with the presence of AVS for the first time in the literature. MHR showed the best associational strength in AVS than other inflammation hematological ratios such as SII, NLR, PLR and LMR.

## Data Availability

The original contributions presented in the study are included in the article/Supplementary Material, further inquiries can be directed to the corresponding authors.
